# Path Planning for Autonomous Mobile Robots: A Review

**DOI:** 10.3390/s21237898

**Published:** 2021-11-26

**Authors:** José Ricardo Sánchez-Ibáñez, Carlos J. Pérez-del-Pulgar, Alfonso García-Cerezo

**Affiliations:** Space Robotics Laboratory, Department of Systems Engineering and Automation, Universidad de Málaga, C/Ortiz Ramos s/n, 29071 Málaga, Spain; carlosperez@uma.es (C.J.P.-d.-P.); ajgarcia@uma.es (A.G.-C.)

**Keywords:** guidance, autonomy, vehicle, survey, trajectory, route, graph search, sampling, wheeled

## Abstract

Providing mobile robots with autonomous capabilities is advantageous. It allows one to dispense with the intervention of human operators, which may prove beneficial in economic and safety terms. Autonomy requires, in most cases, the use of path planners that enable the robot to deliberate about how to move from its location at one moment to another. Looking for the most appropriate path planning algorithm according to the requirements imposed by users can be challenging, given the overwhelming number of approaches that exist in the literature. Moreover, the past review works analyzed here cover only some of these approaches, missing important ones. For this reason, our paper aims to serve as a starting point for a clear and comprehensive overview of the research to date. It introduces a global classification of path planning algorithms, with a focus on those approaches used along with autonomous ground vehicles, but is also extendable to other robots moving on surfaces, such as autonomous boats. Moreover, the models used to represent the environment, together with the robot mobility and dynamics, are also addressed from the perspective of path planning. Each of the path planning categories presented in the classification is disclosed and analyzed, and a discussion about their applicability is added at the end.

## 1. Introduction

Autonomous navigation is a valuable asset for mobile robots. It helps to mitigate their dependency on human intervention. However, it also entails many tasks or problems to solve, e.g., path planning. This task lies in finding the best course of action to make a robot reach the desired state from its current one. For example, both states could be, respectively, the goal and the initial position. This course of action comes in the form of a path, also named a route in many other works. The path serves to guide the robot to the desired state in question. However, there may be numerous possible paths, given the free space in which the robot can move. Path planning algorithms generally try to obtain the best path or at least an admissible approximation to it. The best path here refers to the optimal one, in the sense that the resulting path comes from minimizing one or more objective optimization functions. For instance, this path may be the one entailing the least amount of time. This is critical in missions such as those of the search-and-rescue field [1]: victims of a disaster may ask for help in life-or-death situations. Another optimization function to consider could be the energy of the robot. In the case of planetary exploration, this is critical since rovers have limited energetic resources available [2]. At the same time, the path generated by the planner must follow any imposed restrictions. These may come from the limitations in the adaptability of the robot to certain terrains. The locomotion of the robot and the characteristics of the existing terrain limit the kind of manoeuvres that can be performed. This consequently reduces the number of feasible paths that the path planner can generate.

In the literature there are a vast number of path planning approaches and this number has continued to increase over the years. For this reason, selecting the most appropriate approach given certain requirements (for instance, the aforementioned locomotion restrictions) can be a challenging task. Moreover, as discussed below, the latest reviews and surveys on path planning do not offer a comprehensive overview of the majority of existing path planning solutions. This is the main motivation for writing this review paper: it describes in detail different path planning categories and, for each of them, introduces relevant representative references found in the literature, focusing on those algorithms aimed at robots that move on top of surfaces (ground, water, etc.). This paper is organized as follows. Section 2 presents the method proposed in this paper to classify the existing path planning algorithms. It also makes clear, following this method, the fact that previous works have important omissions. Moreover, this section also provides an analysis of the methods used to address the environment and locomotion information. The next sections each deal with one of the categories of this classification: *Reactive Computing* (Section 3), *Soft Computing* (Section 4), *C-Space Search* (Section 5) and *Optimal Control* (Section 6). Finally, Section 7 summarizes the contents of this paper and presents a discussion about the path planning algorithms contained in the aforementioned categories.

## 2. Path Planning Algorithms

**Figure 1:**
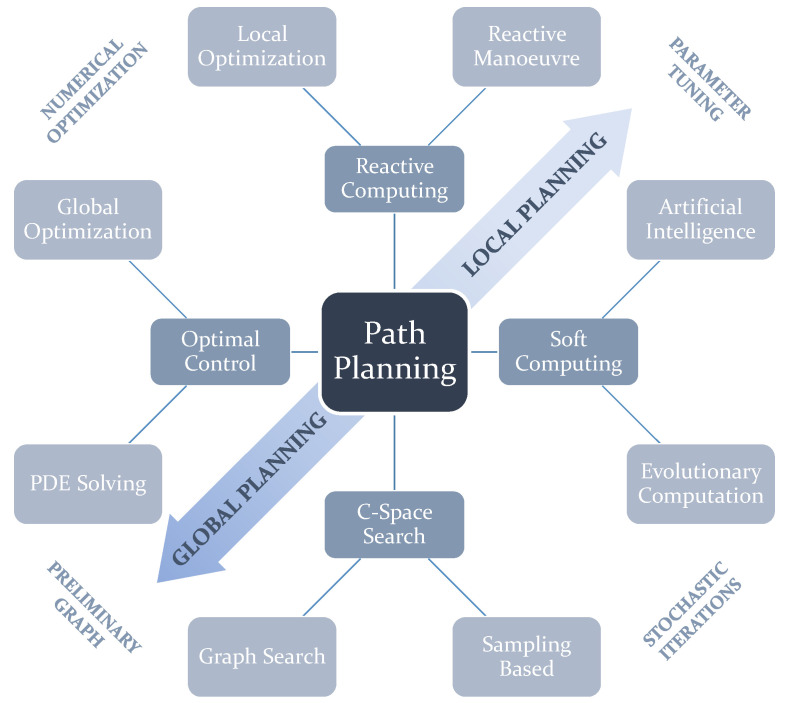
Schematic showing the proposed classification of existing path planning approaches. There are four main categories, each of them containing two subcategories. Two adjacent subcategories from different categories have features in common. The schematic also indicates how some subcategories are more inclined towards either *Global Planning* or *Local Planning*.

Figure 1 depicts the four mentioned categories of path planning, splitting each of them into two subcategories. This classification rests on the principles and fundamental mechanisms used to construct and return a path. A more detailed insight into these categories and why this classification is arranged in this way is provided in the next subsection. A second subsection presents the different approaches taken in modelling the environment and the robot–terrain interaction. We considered it necessary to add this as for many algorithms, especially those in the *C-Space Search* category, it is necessary to construct these models beforehand.

### 2.1. General Classification

The proposed classification considers how path planning algorithms function. In many past reviews, two kinds of distinction were made: according to whether the environment is dynamic or not; *Online* and *Offline* path planners. respectively [3]; and the size of the environment, whether *Local* or *Global*. Usually, *Online* is associated with *Local* and *Offline* with *Global*. The main issue with this is that there are algorithms that can be considered in both categories. An algorithm with no replanning capabilities could be used online due to its high computational speed. The contrary could also happen. For instance, a *Reactive Computing* algorithm called the Dynamic Window Approach (DWA), is usually used for local planning [4], but can also be used for global planning [5]. Vagale et al. [6] presents an interesting division between algorithms that require a preliminary map representation (*Classic*) [7] and those which do not (*Advanced*). *Classic* includes *Graph Search* methods, whereas *Advanced* addresses *Soft Computing* and *Sampling-Based* algorithms. Souissi et al. [7] proposes several clear and reasonable path planning classifications: according to the robot model (holonomic, non-holonomic, kinodynamic); according to the map model requirement (needed or not needed beforehand); according to the replanning capability (offline or online); and according to whether the algorithm always calculates the same solution or not, according to preliminary configuration parameters (deterministic or probabilistic).

The main purpose of the classification proposed in this paper, depicted in Figure 1, is two-fold. First, this classification aims to encompass a larger variety of algorithms than those that are tackled in past reviews. Many past reviews propose or claim to present a general overview on path planning, but as can be seen in Table 1 the majority of them suffer from important omissions. In this table, *Yes* means there is significant discussion about the algorithms in question. *Only Mentions* means the publication acknowledges the existence of at least one or more algorithms in that class. If there are one or two algorithms between parentheses, this means those are only mentioned/referred to briefly. Second, the nomenclature to refer to path planning categories is not clear in some cases. For instance, some other reviews make a distinction between *Classical* and *Heuristic* approaches [8,9]. Patle et al. [10] refer to the latter as *Reactive*. However, the term *Classical* can be quite an ambiguous term as the majority of planning algorithms used are based on methods with one or more decades of life. This class is also used to encompass many algorithms with completely different ways of functioning. The term *Heuristic* has not only been used to refer to *Evolutionary* and *Artificial Intelligence* algorithms [8], but it has also been also used to refer to *Graph Search*-based planners [4]. With this in mind, we propose the use of a general classification using four classes (see Figure 1): *Reactive Computing*, *Soft Computing*, *C-Space Search*, and *Optimal Control*. Moreover, Figure 1 shows how in general terms each of these categories tend to be used mostly for either *Local* or *Global planning.* Moreover, each of the subcategories can also have something in common in their functioning with subcategories from other categories, such as the use of numerical methods, the existence of parameters to tune the algorithm beforehand, the requirement of modelling the map with a graph or the use of stochastic iterative processes.

### 2.2. Path Planning Workspace Modeling

A path planner needs to be fed with information describing the environment. This information can describe, for example, the presence of obstacles or the features of the surface that are relevant to the planning. In addition, the criteria used to calculate the path has to do with the way the robot interacts with this environment. For instance, merely to minimize path length and perhaps acquire the information as to what areas can be traversed or not is enough, whereas to minimize energy the terramechanics and the way the robot steers should be taken into account.

#### 2.2.1. Environment Modeling

**Figure 2:**
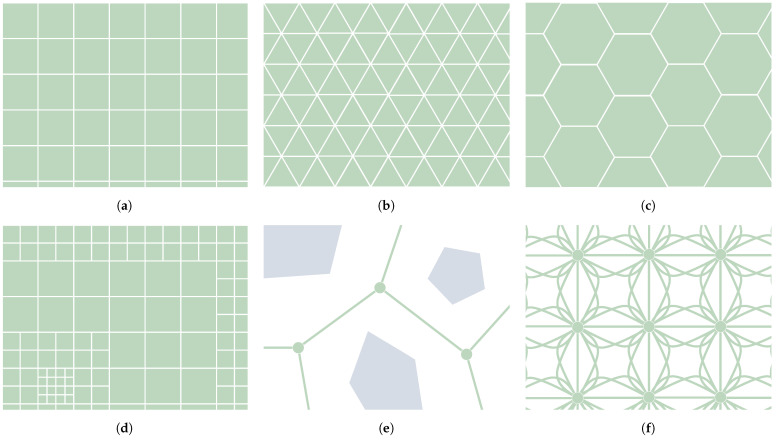
Different types of environment cell decomposition (**a**–**d**) and roadmap graphs (**e**–**f**). (**a**) Tessellations using squares. (**b**) Tessellations using triangles. (**c**) Tessellations using hexagons. (**d**) Irregular grid. (**e**) Voronoi roadmap. (**f**) State lattice graph.

Surface mobile robots drive from one position to another within a certain region in space. Therefore, it is necessary to consider how the locomotion model will interact with this surface and how the path planner will take care of it. For instance, some algorithms require the construction of a graph that somehow represents the environment in which the robot is moving. This is mostly the case for *Graph Search* algorithms, part of the *C-Space Search* category. *Evolutionary* algorithms such as Ant Colony Optimizers (ACO) can also make use of a graph. This asset can represent how the terrain features that affect the robot navigation are spatially arranged in the scenario. In particular, the graph in question is assumed here to be built upon metric maps, acknowledging the existence of other kinds of maps outside of the scope of this paper, such as topological and semantic maps [22]. According to Souissi et al. [7] there are multiple ways to build a graph, as shown in Figure 2. The work of Nash and Koenig [11] has also shed some light onto this classification. It distinguishes between *Cell Decomposition* and *Roadmaps*. The first of these consists of tessellating the surface into cells. These cells can be arranged using regular [3,4,7,11] or irregular grids [7,11]. Figure 2a–c show how regular grids can be built using one out of three types of polygons: squares, triangles and hexagons. Its main advantage is the simple indexation of each node, which is translated into quick access to any of them and an optimized way to store them in memory [23]. Irregular grids, such as the one depicted in Figure 2d, allow the better adaptation the grid to terrain features with different values of resolution, at the expense of possibly obtaining worse paths [24]. Other forms of *Cell Decomposition* are the *Navigation meshes* and *Circle-based waypoint graphs*, as explained by Nash and Koenig [11]. The other form of representation of the environment is, as mentioned, accomplished using roadmaps. A roadmap is a graph built upon nodes connected by edges. Each node represents a possible state of the robot, whereas each edge indicates how to reach that state from another. Examples of roadmaps include Voronoi graphs [25] (see Figure 2e), Visibility graphs [26] and State-Lattice graphs (see Figure 2f). The latter consist of making the edges based upon motion primitives, so the resulting path is ensured to be feasible given the robot mobility constraints, especially when using *Graph Search* algorithms, as in the work of Likhachev and Ferguson [27], Bergman et al. [28].

The cells or the nodes from these graphs can store information regarding the surface at their location, in the form of static or dynamic elements [10]. This can be, for instance, elevation information. A Digital Elevation Map (DEM) is a grid of which the nodes have associated with each of them a value of elevation. Elevation maps can be also represented by polygons, but regular grid maps are preferred [29]. Shape-related features, such as the slope or the surface roughness, can be extracted using convolution matrices [30]. The size of the kernel and the DEM resolution will determine what kind of features are extracted. Moreover, this resolution defines the level of detail of the elements contained in the map. As shown in Figure 2d, this resolution can be non-uniform or multiple. The size of the grid can be chosen according to the scale at which the planning is performed: *Local* in the case of covering the immediate surroundings of the robot (more or less the reachable distance of the on-board sensors) and *Global* if the area is bigger than that, usually using information from external sources such as satellites or drones.

With regards to how the cost is defined over the planner workspace, there are different ways. First of all, we understand cost as the metric that the robot accumulates by moving. The objective of the path planner is to minimize this accumulation by producing the optimal path. The cost in question can be uniform, in the sense that the regions that can be accessed by the robot always have the same value. This approach can be used for collision-avoidance path planning, in which metrics such as the path length in a 2D plane are minimized. Non-uniform cost maps can be used to assign different values of cost to different accessible areas. It can be useful to, for example, define the energetic performance of the robot at each location. Moreover, the cost can be also defined according to a direction vector. This means that the robot will experience different values of cost depending on its heading. In this case, the cost is categorized as *anisotropic* [31], whereas in the contrary case the cost is *isotropic*. Furthermore, the steering manoeuvre of the robot can also have different values of cost according to its locomotion. Finally, it is worth noting that the environment can be fully known, partially known or even fully unknown, requiring for the latter two a planning strategy that is capable of replanning when this knowledge is updated.

#### 2.2.2. Robot–Surface Interaction Modeling

**Figure 3:**
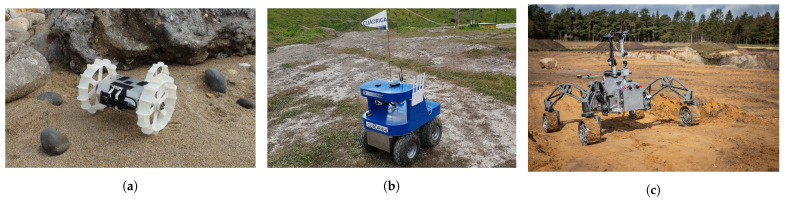
Examples of ground mobile robots with different kinematic configurations. The *Koguma* rover (**a**) has *Differential Drive* locomotion. *Cuádriga* (**b**) is a *Skid-steering* robot owned by our institution, the University of Málaga. *SherpaTT* (**c**) has four steerable wheels that allow it to execute *Full Ackermann*, *Crabbing* and *Point Turn* maneuvers. (**a**,**c**) have been reproduced with permission of the University of Tohoku and the German Research Center for Artificial Intelligence (DFKI), respectively.

**Figure 4:**
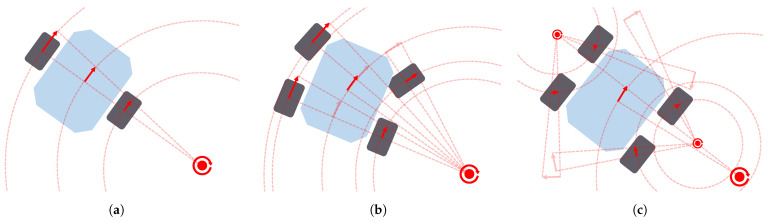

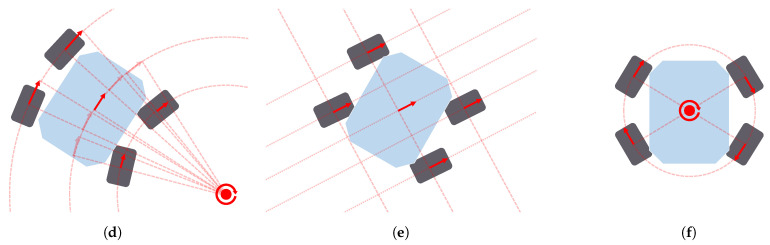
Locomotion models used for wheeled ground mobile robots along with path planners: Differential drive (**a**), Front Ackermann (**b**), Skid-steering (**c**), Full Ackermann (**d**), Crabbing (**e**) and Point Turn (**f**).

A ground mobile robot interacts with the surface beneath it to propel itself. To perform this function, there are many different locomotion actuators, such as wheels, tracks, legs and even omnidirectional wheels. Figure 3 depicts three real examples of ground mobile robots using different configurations of actuators. These actuators, together with the joints linking them to the robot body, determine the kinematic structure and the dynamic behavior of the robot. In other words, they determine the locomotion configuration of the robot. Zhang et al. [20] summarize some kinematic and dynamic models of different well-known configurations: *Differential drive* [32] (see *Koguma* robot [33] in Figure 3a as an example and the depiction of the model in Figure 4a), *Ackermann steering* [34] (see Figure 4b,d), *Skid steering* [35] (see Figure 4c) and *Omnidirectional* [36]. Some of them entail constraints relevant to path planning, such as the minimum turning radius of robots with *Front Ackermann steering* [37] (see Figure 4b) or the high energy consumption of *Skid-steering* robots in turning manoeuvres [38]. Moreover, another model exists, called *Crabbing* (see Figure 4e). It allows a robot to drive in a direction different to the one it is facing, due to having steering joints on top of all wheels [39]. Furthermore, some kinematic configurations allow the robot to perform the *Point Turn* manoeuvre, which makes them rotate without translating. It is worth mentioning the existence of articulated robots that are capable of reconfiguring themselves to obtain some kind of benefit and perform multiple types of locomotion (see *SherpaTT* in Figure 3c as an example of this). For instance, articulated robots with tracks can actively control their stability while driving on rough terrains [40,41]. Others use a wheel-on-legs configuration to execute a locomotion mode called *Wheel-walking* [42,43]. This mode is designed to overcome soft terrains in which a robot could get stuck. In a similar way, *Push-pull* locomotion imitates the motion of a caterpillar to increase traction in this kind of terrain [44,45,46]. Path planning algorithms acknowledging this reconfiguration capability are a must-have for this kind of robot, as they can find paths that take advantage of their high adaptability. In the case of global planning, Rohmer et al. [47] used a *Graph Search* algorithm, Dijkstra, to first produce a path and later, via simulation tools, evaluate which locomotion mode is better to drive each of its parts. We, the authors of this review publication, proposed the use of a *PDE Solving* method to consider the multiple locomotion modes at the time of planning using an isotropic cost function [48,49]. To the authors’ knowledge there are not many existing *Local Planning* approaches addressing the kinodynamic constraints of robots with multiple locomotion modes. Reid et al. [50] proposed the use of a *Sampling-Based* algorithm, the Fast Marching Tree (FMT*), to tackle the motion planning of a reconfigurable hybrid robot with wheeled-legs.

The robot’s locomotion will adapt better or worse depending on the terrain features, which were briefly mentioned before. These features may be related to either the morphology (shape) or the composition of the terrain. One of them is the terrain inclination or slope [51]. The slope has an influence on the *Roll* and *Pitch* orientation angles of the robot, which is important to consider to preserve stability [41,52,53]. Moreover, it can also influence the energetic performance of the robot according to its direction. This dependency on direction is due to the effect of gravity, making the robot consume different amounts of energy according to whether it is climbing, descending, going laterally or going diagonally through the slope [29,54,55,56,57]. Another relevant terrain feature is the roughness. This is the measure of how diverse the normal vectors are [58,59] and may affect the vibration experienced by the robot. Other terrain shapes can be negotiated by the robot, according to its chassis. For instance, it can overcome rocks using the body clearance, which is the space between the body lower surface and the terrain surface [60,61]. The presence of negative obstacles such as holes or ditches can be problematic as they are difficult to capture by the robot’s sensors [62]. With regards to the terrain composition, it has an influence on the dynamics underlying the robot–surface interaction. This surface may be more rigid or deformable [63]. This affects the way the robot adheres to the surface, even restricting its motion [64]. The slippage is the metric that, in general terms, measures how the real speed of the robot differs from the commanded one, usually by calculating a ratio between them [65,66]. Some works consider both slippage and the slope of the terrain to make a more accurate estimation of the robot while traversing rough terrains [67,68]. The magnitude of gravity can also affect the dynamics of the interaction between the robot and the terrain [30,69]. Finally, path planners usually make use of cost functions that encompasses multiple terrain features related to shape and composition. For instance, Ishigami et al. [2] introduced the *dynamic mobility index*, encompassing stability, slippage, elapsed time and energy consumption. Moreover, there may exist other elements not directly related to the terrain that may still affect the performance of the robot while navigating. One of them is the solar radiation [66,70], which can be modeled as a dynamic function [71]. Groves et al. [72] mapped other kinds of radiation that may harm the robot, as in scenarios of nuclear dismantlement. Moreover, the concept of risk can be of high importance to prevent the robot from getting into a dangerous situation [73], such as increasing the cost with the proximity to obstacles [74].

## 3. Reactive-Computing-Based Path Planning Algorithms

This category encompasses path planning algorithms where the environment, usually a map distinguishing between obstacle and non-obstacle regions, only indicates the location and shape of the existing obstacles. *Reactive Computing* algorithms are usually employed as local path planners (covering the surroundings of the robot and with dynamic replanning) due to their capability to quickly handle new information (e.g., in the form of newly discovered obstacles), which often comes from the limited onboard sensors. As local planners, these algorithms usually plan the next immediate path or manoeuvre to avoid nearby obstacles while following a global plan made by another algorithm. However these algorithms may calculate local minima paths, or even cause the robot to get stuck, so special care must be taken. There are two subcategories of *Reactive Computing* algorithms: *Reactive Manoeuvre* methods, where the presence of obstacles determines the immediate next manoeuvre of the robot, and *Local Optimization* methods, where an existing path is modified according to the presence of obstacles.

### 3.1. Reactive Manoeuvre

The algorithms presented here rely on defining how the robot reacts at each instant to the presence of obstacles. This reaction can be defined according to a formulation that addresses the location of existing obstacles. A common feature of the different formulation approaches is the low computational requirements needed to produce the reaction, usually in the form of a steering or velocity command. Since this formulation lacks global information, these techniques are commonly used as *Local Planners*. The formulation in question may be based on the use of fields to tackle the location of obstacles, the detection of obstacle boundaries to circumvent them, or the production of a velocity command after evaluating the available free space or the speed of moving obstacles.

**Figure 5:**
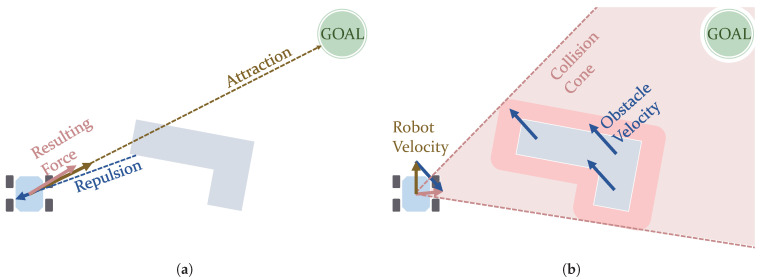
Graphical representations of concepts used in Artificial Potential Field (**a**) and Velocity Obstacle (**b**) algorithms. (**a**) Potential fields acting on a robot. (**b**) Collision cone considering a moving obstacle.

The use of field methods include the Artificial Potential Fields (APF) and the Vector Field Histogram (VFH) algorithms. In APF, the motion of a robot can result from the sum of virtual forces that external elements such as obstacles create. In this way, the robot gets further from these obstacles and avoids colliding with them, as the forces from them are repulsive [75]. An attractive force created by the target position makes the robot go towards it. This can be seen in the picture displayed in Figure 5a. Ge and Cui [76] presented an application of APF in environments containing dynamic obstacles. However, the main drawback of this strategy is that it is prone to causing the robot to get stuck in local minimum points. For this reason, further research works were oriented to overcome this issue. This was the main target of the work by Vadakkepat et al. [77]. They combined APF with genetic methods (*Evolutionary* algorithms) to overcome this situation. This combination was also used to plan the motion of a simulated 6-wheel rover [78]. Another hybrid version includes the use of PSO algorithms [79]. Triharminto et al. [80] proposed a non-hybrid solution, consisting of adding a repulsive potential field around the rover. However, it has not yet been tested with U-shaped obstacles. Other works solve this issue by creating custom escape paths whenever it is detected that the robot is on a local minimum point [81]. Furthermore, Bayat et al. [82] present a solution inspired by electrostatic potential fields in which, instead of using a sum of virtual forces, a so-called scalar potential field guides the robot. VFH, proposed by Borenstein et al. [83], creates a polar histogram to evaluate the density of obstacles around the robot, selecting the steering angle with the lowest density of obstacles. Some improvements to VFH were introduced years later [84,85].

**Figure 6:**
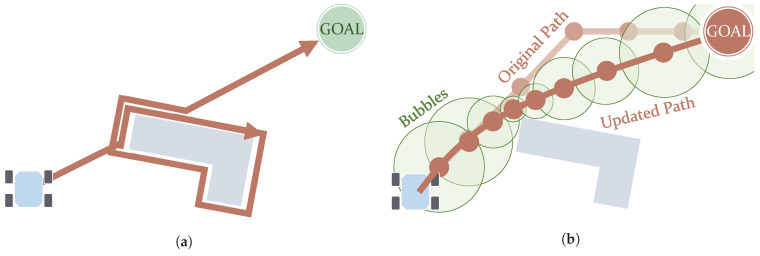
Graphical representations of concepts used in the Bug algorithm (**a**) and Elastic Bands (**b**) algorithm with Bubble bounds. (**a**) Path after using the Bug1 algorithm. (**b**) Path after experiencing stretching.

The Bug algorithms, Bug1 and Bug2, make the robot circumvent any obstacle found on its way until it reaches the goal [86]. The main difference between them is that Bug1 makes the robot drive the full boundary of any obstacle (see Figure 6a), whereas Bug2 can drive it only partially [19]. They despise optimality in favour of easiness in the implementation and very minimal computation. They can be used on robots equipped only with sensors that just detect obstacles in their immediate vicinity. In this way, these robots either drive towards the goal or drive along the boundaries of obstacles they find. The works of Buniyamin et al. [87] and Campbell et al. [19] refer to some variants that improve this kind of algorithm, in general reducing the distance the robot drives. Xu et al. [88] uses the Bug algorithm considering turning radius constraints, producing paths with smooth turns.

The following approaches are focused on producing a velocity command for the robot. Velocity Obstacles methods calculate a safe trajectory considering the velocity vectors of both the robotic agent and any other moving obstacle [89,90]. This calculation evaluates a cone of collision such as the one shown in Figure 5b. Chen et al. [91] used Velocity Obstacles in an hybrid fashion with another algorithms called Fast Marching Square (FMS). Wilkie et al. [92] considered the *Front-ackermann* (see Figure 4b) locomotion model for cars. Chakravarthy and Ghose [93] proposed using a Collision-cone algorithm, similar to Velocity Obstacles but considering obstacles with arbitrary shapes. Qu et al. [94] introduced its use along with car models (with *Front-ackermann* configurations, as seen in Figure 4b). Finally, the Dynamic Window Approach (DWA) is an algorithm that searches in the velocity space for a velocity command to follow a collision-free circular trajectory, delimited by admissible speed values and a time window [95]. This solution may not be the globally optimal one, but rather a locally optimal one [4]. DWA can be used even for robots navigating at high speeds [96]. Feng et al. [97] propose an improved version that reduces its complexity by reducing the velocity space to search. Other approaches use this algorithm for energy-minimization path planning [98,99]. Although commonly used as a local planner, Zhang et al. [5] have proposed its use at a global scale as a global path planner.

### 3.2. Local Optimization

These algorithms usually start from a pre-existing path and modify it according to the existing obstacles. Here it is prioritized to keep computational use to the minimum at the expense of losing optimality or even completeness. There are different options to modify the path, ranging from the selection of velocity profiles within a velocity space to the stretching and elongation of the path under the effect of artificial forces.

The use of Elastic Bands in path planning was proposed by Quinlan and Khatib [100]. This method deforms an existing collision-free path according to the obstacles, stretching (see Figure 6b) or elongating it. From a set of points in this path, a set of overlapping subregions, called Bubbles, is created. These Bubbles cover collision-free areas and their size is bounded by the distance to obstacles. This entails that smaller and more numerous Bubbles are present in the portions of the path closer to obstacles. It can be used in dynamic environments, although large changes may lead to the failure of the method [100]. Moreover, it has also been adapted to non-holonomic vehicles by complying with curvature constraints Khatib et al. [101] and using *Bezier* curves [102]. An extension to Elastic Bands includes time constraints and is named Timed Elastic Bands (TEB). This extended version addresses the kinodynamic constraints of the robot [103].

## 4. Soft-Computing-Based Path Planning Algorithms

This kind of algorithm does not intend to find the exact optimal solution, but rather to approximate it, tolerating a certain range of imprecision. In general, these algorithms require the tuning of certain parameters by the user in order to work properly according to the characteristics of the environment. They can deal even with dynamic environments and are adequate for problems involving a large number of variables and high degrees of freedom [8]. However, in general they demand a high number of computational resources. This review follows the classification proposed by Mirjalili and Dong [104], which distinguishes between *Evolutionary*, *Fuzzy control* and *Machine learning* methods. The first one uses techniques inspired by biology and nature: they start with a system formed by individuals that changes over time, i.e., evolves. *Fuzzy control* and *Machine learning* methods are here part of a subcategory named *Artificial Intelligence*. They use fuzzy rules and neural networks, respectively, to produce controllers. These controllers are very useful for navigating through initially unknown scenarios and in general produce paths according to the obstacles the robot detects on its way. To sum up, *Soft Computing* algorithms allow the tuning of a series of repetitive elements, either nature-based individuals, fuzzy rules or artificial neurons, to generate a path.

### 4.1. Evolutionary Computation

*Evolutionary* algorithms are also known as *Meta-heuristic* or Nature-inspired [105]. These algorithms generate a path that results from the evolution of a population. This population is made up of intelligent individuals whose actions are modelled after behaviors found in nature [104]. These actions may involve modifying themselves and/or interacting with other individuals. In some cases, these operations imply a motion by the individuals in the free space of the environment, i.e., in the space reachable by the robot. After performing a series of these operations, the algorithms approximate the optimal solution. The resulting path and the time invested to converge depend on the behavior policy assigned to the individuals, the nature of the scenario and the values assigned by the user to certain configurable parameters. An example of the latter is the number of individuals that populate the path planning problem. *Evolutionary* algorithms include *Genetic* methods and *Swarm Optimizers*. The first method uses chromosome models, whereas the second one models the behavior of living beings.

**Figure 7:**
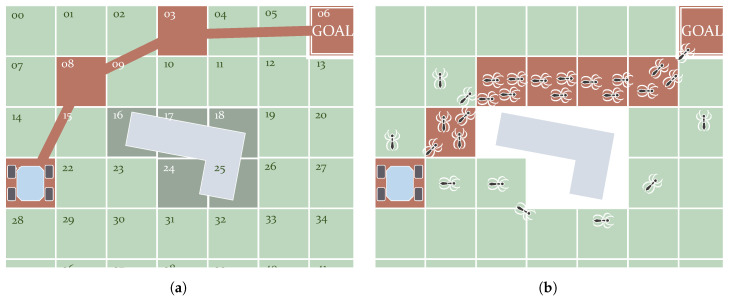
Examples of *Evolutionary* algorithms: *Genetic* (**a**) and *Swarm Optimizer* ACO (**b**). (**a**) Functioning of the genetic algorithms used to perform path planning. The path in this figure has the form of a chromosome with genes 21–08–03–06. (**b**) Functioning of the ACO algorithm. Simulated ants deposit more pheromones in the shortest path. Eventually the majority will follow this path.

As just mentioned, *Genetic* algorithms work with individuals modeled after chromosomes [106]. These individuals contain genes, as chromosomes do, in the form of binary numbers. These numbers encode a solution, which is the set of *waypoints* forming a path in the particular case of solving the path planning problem. In other words, each chromosome in the population represents a path. Figure 7a depicts an example using a grid. This grid is made up of cells, each of them labeled with a number. The genetic algorithm starts with a random set of chromosomes. This set evolves using three processes: *Reproduction*, *Crossover* and *Mutation* [107]. *Reproduction* creates new chromosomes by copying the best ones. It also removes the worst ones. *Crossover* is the process in which chromosomes interchange their genes. *Mutation* introduces random changes in the genes to incentive the exploring of the search space and to avoid local minima. As a result of continuously repeating these processes, the algorithm converges. Zhang et al. [4] mention *Genetic* algorithms that converge more slowly as they get closer to the optimal solution. Han et al. [108] used *Genetic* algorithms to find the shortest paths in environments with dynamic obstacles. Tuncer and Yildirim [109] presented a modification to the *Mutation* process. This consists of checking free nodes around the position that is about to be mutated. This work compares the results with previous variants of the method. Another research work used genetic algorithms, along with large grids up to 2000 × 2000 nodes [110]. Genetic algorithms for path planning were also tested on an experimental platform [111]. More improvements include the initial selection of *waypoints*, considering those located near obstacles [112]. Elhoseny et al. [113] introduced a more regulatory policy on exploration during *Mutation*, taking into account how diverse the chromosomes are. Furthermore, this work not only optimized path length but also preserved smoothness using *Bézier* curves. Finally, the *Crossover* process is also improved upon in the work of Lamini et al. [114] to make the solution converge faster and also to reduce the number of turns made by the robot. There are many path planning applications based on *Genetic* methods presented in the review of Patle et al. [10].

Unlike the algorithms based on *Genetic* methods, *Swarm Optimizers* use agents that move and actuate in the free space. These individuals are modeled after animals in most cases. After a series of iterations, the motion of these individuals towards the goal creates a pattern that eventually converges to the resulting path. Table 2 introduces some of the models used that can be found in the literature. The Particle Swarm Optimization (PSO) algorithm stands out because of its simplicity. It is inspired by the behavior of certain groups of animals such as fish schools [115]. It creates a series of particles that relocate themselves over time until the algorithm converges. These algorithms search for the best positions and communicate with each other, considering their previous experience [116]. Mac et al. [117] propose a path planner that combines PSO with the Dijkstra algorithm (a *Graph Search* planner that is discussed below). Another well-known algorithm is the Ant Colony Optimizer (ACO) which, as the name indicates, simulates the behavior of ants. These insects move while leaving a trail of pheromones in their search for food. This trail can be tracked by the rest of the ants. Those places that contain more pheromones make up the *waypoints* of the best-found path. Figure 7b depicts this concept in a situation with an obstacle between the start and goal positions. Here, the best path is the shortest one. Following the same strategy, virtual ants can move on a grid, leaving more or fewer pheromones according to their state concerning the goal [118,119]. To avoid falling into a local minimum, some works combine this method with heuristic functions [120,121]. Che et al. [122] also solve this by adopting a rule used in the Grey Wolves approach. Luo et al. [123] present some improvements for ACO to not only avoid deadlocks but also to reduce the time it needs to converge. This algorithm has also been simulated with DEMs to minimize time [124] and energy [125]. The latter has also been achievedwhile avoiding dynamic obstacles, which is also addressed by the work of Sangeetha et al. [126]. This work combines ACO with fuzzy control. There are plenty more nature-inspired approaches. To avoid extending too much, some of them are indicated in Table 2. Moreover, there are also cases in which two models are combined. This is the case of the approach presented by Saraswathi et al. [127], where a hybrid between Cuckoo and Bat algorithms is tested.

### 4.2. Artificial Intelligence

*Soft Computing* algorithms may use other sets of configurable operators such as fuzzy rules or neural networks. Seraji and Howard [140] demonstrate the use of fuzzy logic on an experimental mobile platform to navigate through unstructured terrain. Zavlangas and Tzafestas [141] provided a fuzzy-logic-based system designed to make a mobile robot autonomously navigate through a dynamic environment, avoiding obstacles on its way. The work of Wang et al. [142] focuses on preventing the robot from getting stuck in local minimum points, such as those produced by U-shaped obstacles. Pandey et al. [143] also provides the results of simulation tests of fuzzy logic for obstacle avoidance. With regards to the use of neural networks, Yan and Li [144] also use fuzzy logic on a platform focusing on minimizing computational resources and traversing environments containing dynamic obstacles. Pandey and Parhi [145] combines fuzzy logic and a population-based algorithm named Wind Driven Optimization that tunes the fuzzy rules. With regards to the use of neural networks, Zou et al. [146] presented a brief survey on this kind of algorithm for applications at the beginning of the 2000s. Engedy and Horváth [147] presented a path planner using neural networks for mobile robots that must avoid static and dynamic obstacles. Zhang et al. [148] used this kind of technique to find the shortest path in maze scenarios. This approach has also been used together with genetic algorithms [149,150].

Other works in the literature combine fuzzy logic and neural networks [151,152,153,154]. There have been different approaches to this. For instance, Mohanty and Parhi [155] use many of these systems for autonomous navigation. Further insights and a more extensive surveys on related hybrid methods are provided in Mac et al. [8]. Furthermore, the use of Reinforcement Learning (RL) has also been studied to control the motion of a robot [156,157]. Faust et al. [158] combined RL with the Probabilistic Roadmap Method (PRM), which is one of the algorithms detailed next. For more information about planning algorithms based on RL, refer to the work of Sun et al. [21].

## 5. C-Space-Search-Based Path Planning Algorithms

Algorithms in this category consider the working space of the path planner as the space of all states or configurations reachable by the robot. For this reason, most of the works in this category refer to this working space as the *C-Space*. The main idea behind these algorithms is to use a discrete set of samples that are part of this *C-Space*. In other words, the *C-Space* is discretized. This set of samples includes the initial and the goal states, or at least samples relatively close to them. In this way, these algorithms execute a search operation, visiting samples from this set. At a certain point, the algorithm will find and return a certain subset of samples connecting the initial and goal states representing the resulting path. In other words, the *waypoints* forming the paths correspond, each of them, to a sample from the C-Space. This implies that the generated path heavily depends on how these samples are scattered, how they are connected and how are they visited. In fact, due to this dependency, in some approaches post-processing is done to smooth the shape of the resulting path.

The *C-Space Search* category is subdivided into two groups of algorithms according to how they discretize the *C-Space*. *Graph Search* algorithms do this using a pre-existing graph (such as one of those depicted in Figure 2). Each of the nodes from this graph represents a *C-Space* sample and is connected to other nearby nodes, i.e., its neighbors. With regards to *Sampling-Based* algorithms, they focus on the creation and/or modification of samples within the *C-Space* in an iterative way. They can keep working, even after finding a feasible path, to find better ones.

### 5.1. Graph Search

**Figure 8:**
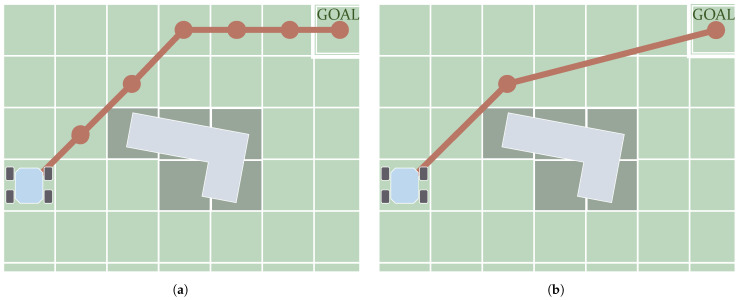
Main difference between the paths (in red) produced by *Edge-restricted* (**a**) and *Any-angle* algorithms (**b**): in the first case, *waypoints* can be placed only on consecutive (neighboring) nodes.

As mentioned, the *C-Space* can be discretized in the form of a graph. *Graph Search* algorithms fully or partially visit this graph until they find a path connecting the initial and goal states. The first algorithms made in this category return paths of which the *waypoints* are placed on top of neighboring samples. In other words, the connections between consecutive *waypoints* of the path are coincident with the graph edges. Figure 8a depicts a schematic showing how in the first case the shape of the path is determined by these edges. This review, therefore, categorizes the *Graph Search* algorithms generating this kind of path as *Edge-restricted*. These paths consequently depend on how the graph is structured. As seen in Section 2.2.1 there are various graph structures in the form of *Cell Decomposition* and *Roadmaps*. Another kind of *Graph Search* algorithm, *Any-angle*, were created to solve this issue with the restriction to the graph edges. The reason this name is used is that paths produced by *Edge-restricted* planners only use certain values of orientation. For example, in an eight-neighborhood regular grid, as in the case shown in Figure 8a, *Edge-restricted* paths can only have orientations with 0, ±45, ±90, ±135 and 180 degrees. *Any-angle* algorithms produce paths that are not restricted to these orientations as their *waypoints* do not necessarily have to be placed in neighboring nodes. Figure 8b depicts an example of this.

**Figure 9:**
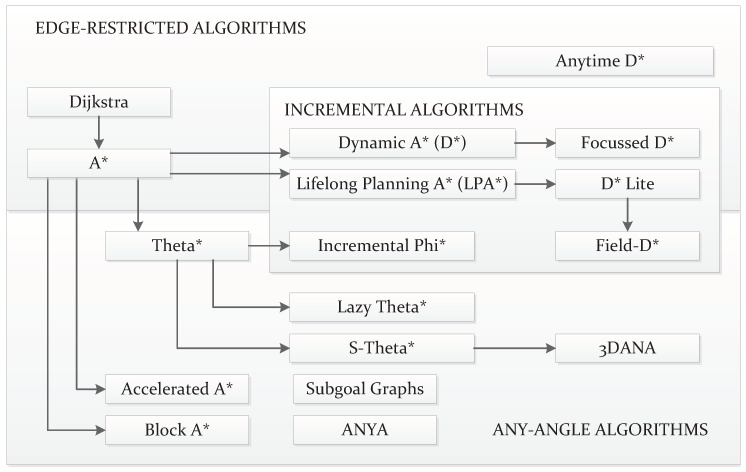
Overview of many different approaches to *Graph Search* path planning. The arrows indicate how most of them rest on older approaches yet introduce significant improvements.

With regards to *Edge-restricted* algorithms, Figure 9 shows a schematic with the most representative versions of them that can be found in the literature. The most known and basic *Edge-restricted* path planner is the Dijkstra algorithm [159]. As an initial step, this algorithm takes one node, either the start or the goal. Thereafter, it proceeds to propagate information to its neighbors. It could be either the value of the cost required to arrive from the start or the one that remains to arrive at the goal. Iteratively, the algorithms visit the neighbors of already-visited nodes. The information about the amount of cost keeps propagating, and the algorithms assign to each visited node a parent node. If the environment allows it, i.e., no obstacles are isolating either the goal or the start, the algorithm ends up visiting both of them. At this point, the path is retrieved by backtracking the parent nodes. In other words, the path starts from the last visited node and goes back through the parent nodes. Years later, Hart et al. [160] implemented a heuristic version, A*, to speed up computation. Later on, further improvements were introduced. D*, also named Dynamic A*, was introduced by [161] as an incremental version of A*. The fact that it is incremental means that this algorithm recycles previous computations whenever there are changes in the cost assigned to the grid nodes. This prevents the algorithm from executing a whole new computation from scratch. This reduction in the computation allows for rapid replanning in cases where the robot encounters novel obstacles on its way, for example. An improved version called Focussed D* managed to further reduce the computation time of D* [162]. Koenig and Likhachev [163] propose the use of Lifelong Planning A* (LPA*) as another direct incremental extension of A*. This was taken into account as a reference to produce a much simpler version of D* called D*-Lite [164,165]. Colas et al. [166] employed this algorithm on mobile robots in search-and-rescue applications. Since A* and D* algorithms, including their versions, make use of heuristic functions, the resulting paths can be sub-optimal. Likhachev et al. [167] propose anytime versions of these algorithms, which use a configurable fixed time. The best path found, at a given time, is generated by these anytime versions. Dolgov et al. [168] propose a version of A*, *Hybrid* A*, that prioritizes the feasibility of the resulting paths in exchange for the loss of optimality and completeness by rearranging the nodes after a path is found; in a way, the path is kinematically feasible.

With regards to the *Any-angle* algorithms, one of the first ones was Field-D* [169]. This is a well-known algorithm mainly due to its use on Mars NASA rovers since *Spirit* and *Opportunity* [170]. Similarly to D* and D*-lite, it is an incremental algorithm, so it recycles previous computations in subsequent executions. Although Field-D* arose as an outstanding method to overcome the problem of paths restricted to edges, there was still a margin to improve the results and find even more optimal paths. Years later, more *Any-angle* algorithms were created having A* as a basis, focused on the problem of finding the shortest paths while avoiding obstacles. Nash et al. [171] created Theta* for this purpose. They presented it in two versions: one computationally cheaper, *Base*-Theta*, and another more expensive but with results closer to the globally optimal shortest path, named *Angle-Propagation* Theta*. The main premise behind Theta* was the consideration of heading changes on obstacle corners, reducing these changes along the path in contrast with Field-D* [172]. An incremental version of the *Base*-Theta* algorithm was later introduced with the name of Incremental Phi* [173]. A faster version of Theta*, *Lazy*-Theta*, was introduced by Nash et al. [174]. Theta* was also improved to work better on non-uniform cost maps, where the cost increases the closer it gets to obstacles [175]. The Accelerated A* algorithm was introduced by Šišlák et al. [176]. It finds shorter paths than Theta* but at a slower rate (although still faster than A*). Yap et al. [177] also introduced another algorithm named Block A* and compared its performance with A* and Theta*. Another comparative study including Accelerated A*, Block A*, Field-D* and Theta* was provided by Nash and Koenig [11], also including variants of the latter algorithm. The same authors later compared this algorithm with the use of visibility graphs [178]. Munoz and R-Moreno [179] proposed the use of S-Theta* to produce paths that smooth the heading changes. 3DANA is used to produce paths on elevation maps [180,181]. Other algorithms that have shown improved results compared to Theta* and many other *Any-angle* algorithms include *Any-angle* Subgoal Graphs [182,183] and Anya [184,185].

### 5.2. Sampling-Based

*Sampling-Based* path planning algorithms create samples of the C-Space one after another, following different policies [12,186]. Later on, they retrieve the path from the created samples after meeting a certain condition or set of conditions, such as reaching a time limit. This kind of algorithm is asymptotically optimal. It means they can create more and more samples, attempting to find a better solution as time goes on. In general, these algorithms are usually used for searches in high-dimensional spaces. However, the number of samples may be relatively large in order to get close to the global optimal solution [14], demanding the use of large memory resources to store all the samples.

**Figure 10:**
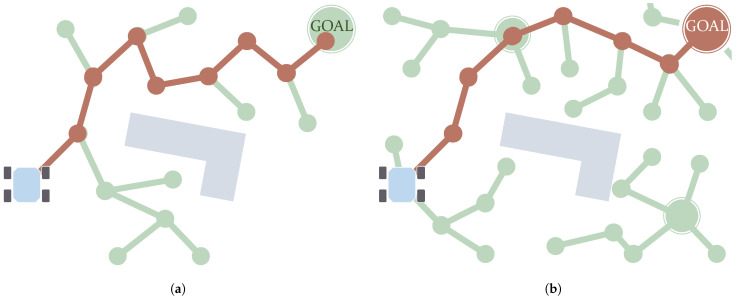
Example cases of single-query (**a**) and multiple-query (**b**) *Sampling-Based* algorithms. Samples are created in an iterative way until the destination is reached.

If only two points are considered (the starting position or state and goal), the algorithm is a single-query algorithm, whereas if more points are selected for the same environment then the algorithm is categorized as multiple-query. With regards to the single-query, one of the most famous is the Rapidly Random Tree (RRT) algorithm, which is also a special case of the Rapidly Deterministic Tree (RDT) [187]. This algorithm emulates a tree growing in the sense that from a starting point the samples are dynamically created as if they were branches. Figure 10a depicts an scheme summarizing this process. When one of the samples is closer to the goal than a certain distance, then the path can be retrieved by tracking backwards until reaching the origin point. As mentioned, more iterations can still be executed to find better paths. Further modifications of RRT can be found in the literature. A bi-directional version was introduced by Kuffner and LaValle [188] with the name of RRT-Connect. Later on, Yershova et al. [189] presented an improved version of RRT called *Dynamic Domain* RRT, which was aware of the obstacles existing in the environment during the expansion of the tree. Arslan and Tsiotras [190] took ideas from the *Graph Search* algorithm LPA to make RRT#, an improved version of RRT with a faster convergence rate. Karaman and Frazzoli [186] introduced a heuristic version of RRT named Heuristic RRT (RRT*) to speed up computation while still being asymptotically optimal. For an extensive review of RRT* variants, refer to the work of Noreen et al. [14]. An improved version called Informed RRT* improved upon the performance of RRT* by delimiting an ellipse enclosing the start and goal positions when a feasible path is found [191]. The next iterations to improve this path are done within this ellipse, instead of making the algorithm explore other options that probably will not influence the result. Gammell et al. [192] presented another improvement to this, called Batch Informed Trees (BIT*), together with a comparison demonstrating its better performance than RRT*, Informed RRT* and FMT*. BIT* also takes some steps from the *Graph Search* algorithm LPA*. Regionally Accelerated Batch Informed Trees (AIT*) improved upon BIT*, especially for cases where narrow corridors exist [193]. The Adaptively Informed Trees (AIT*) and Advanced Batch Informed Trees (ABIT*) algorithms improved upon the BIT* algorithm and were integrated into an experimental NASA rover [194,195].

With regards to multiple-query *Sampling-Based* algorithms, the most famous is the Probabilistic Roadmap Method (PRM) [196]. This algorithm starts with a series of samples that are already scattered over the *C-Space*. From here, new samples are created, creating a new tree from each of these initial samples. Figure 10b illustrates the concept behind this process. Thereafter, a *Graph Search* method such as A* is used to retrieve the path using the graph created by PRM. Karaman and Frazzoli [186] introduced a heuristic version of PRM. The improvement proposed by Park et al. [197] uses a hierarchical structure that reduces the number of samples. PRM has been tested in robots within simulated indoor scenarios in the work of Alenezi et al. [1]. Ichter et al. [198] presented *Critical* PRM, an algorithm that combines PRM with *Reinforcement Learning* to determine critical locations such as narrow corridors.

Another *Sampling-Based* algorithm, named the Fast Marching Tree (FMT*) algorithm, was created to reduce the convergence rate of both RRT and PRM. It takes features from not only the two of them but also an *Optimal Control* algorithm called FMM, which is detailed below. The main objective of FMT* is to find paths, avoiding obstacles, in problems involving a high number of degrees of freedom. One example of this is the motion planning of an articulated vehicle presented by Reid et al. [50]. Ichter et al. [199] propose the use of Group Marching Tree (GMT*), a similar algorithm to FMT* but which focuses on speeding up computation via parallelization using GPUs. Finally, it is worth mentioning there are path planning algorithms that combine the *Dynamic Sampling* approach with Model Predictive Control (MPC) techniques to account for kinodynamic constraints [57,200,201].

## 6. Optimal-Control-Based Path Planning Algorithms

The baseline of *Control Approach* based algorithms is the creation of a control function that takes the robot from an initial state in the C-Space to the destination. As the name suggests, here the path planning problem is addressed using an optimal control approach [202]. The main difference with *Soft Computation* methods is that there are no configurable parameters; the problem here must be fully enclosed. Here, there are two different subcategories. In the first of them, *PDE Solving*, algorithms solve a Partial Derivative Equation (PDE) on a grid, based on the Dynamic Programming Principle (DPP) [203]. The second subcategory, *Numerical Optimization*, encompasses algorithms that in general optimize a pre-existing path given the kino-dynamic restrictions of the robot to make it feasible.

### 6.1. PDE-Solving-Based

The optimal control approach is based here on the Dynamic Programming Principle through the resolution of the Hamilton–Jacobi–Bellman equation [204] using a grid. Since it is a partial derivative equation (PDE), this sub-category is named *PDE Solving*. It can be seen as finding the numerical solution to the problem of calculating the propagation of a wave over a grid. A value of the wave arrival time is assigned to each of the grid nodes. The way the wave propagates will depend on how the HJB equation is formulated, including the cost function. The main drawback of this kind of algorithm is that it generally cannot deal with constraints in the form of discontinuities.

**Figure 11:**
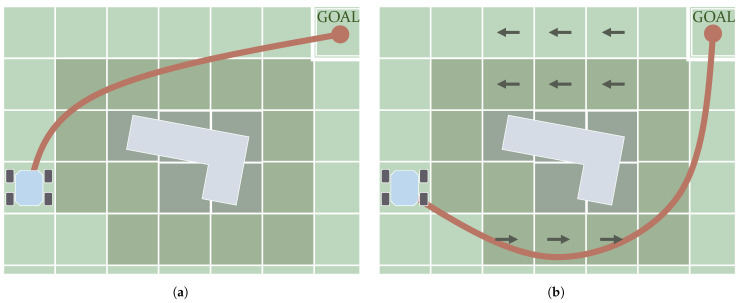
*PDE-Solving*-based algorithms can calculate a continuous and smooth path in non-uniform cost maps. The cost assigned to each cell can be a scalar value or isotropic (**a**) or in the form of a vector, i.e., anisotropic (**b**).

A particular case of the HJB equation is the *Eikonal* equation. This is not only static but also considers the cost function and only returns a scalar value according to the position on the map. This means the wave propagates on a node with a rate that depends only on the assigned scalar value. In this way, the characteristic directions are coincident with the gradient of the *Total Cost* function, and hence the path can be retrieved simply using a *Gradient Descent* method. A whole family of methods have been proposed over the years to compute the solution to this problem-formulation with low computational requirements, and hence they are named *Fast* methods [18]. One of the most well-known is the Fast Marching Method (FMM), introduced by Sethian [205]. This algorithm follows the same strategy as Dijkstra to visit the nodes of a grid. Unlike Dijkstra, FMM assigns the value of an amount of cost to each node by solving the *Eikonal* algorithm. The resulting path is smooth, continuous and optimal. Chiang et al. [206] compared this algorithm with A* and demonstrated how, as the path is not restricted by the grid, FMM gets shorter paths. There are many existing works using FMM for path planning [207,208,209,210]. Some of its variants, most of them reducing the computational power requirements of FMM, are introduced in the review by Gómez et al. [18], along with other *Eikonal* solvers. They are *Binary* FMM, *Fibonacci* FMM, *Simplified* FMM and *Untidy* FMM. The cost values used by the *Eikonal* determine the rate of propagation of the computed wave. Petres et al. [24] demonstrated how the gradient of these cost values affects the curvature radius of the resulting path. Their work also introduced the idea of Heuristic Fast Marching (FM*) and also compares this with other *Graph Search* algorithms. For smooth obstacle avoidance, Fast Marching Square (FMS) [74] computes FMM twice, being the first to create a repulsive field surrounding obstacles. Figure 11a depicts how the path smoothly gets further from the obstacle thanks to the higher values of cost near it (darker colors). Liu and Bucknall [211] used FMS, along with a modification of the cost around the initial position, to consider the initial orientation of the vehicle. Researchers have also aimed to propose incremental versions of FMM, such as E* [212,213,214].

To work with more general expressions of the Hamilton–Jacobi-0Bellman (HJB) equation, other kinds of methods must be used. FMM produces sub-optimal results if used with direction-dependent (anisotropic) costs [215]. This kind of cost implies that the wave propagates differently depending on its direction relative to how a vectorial cost is assigned to the node. There are particular situations in which FMM produces accurate results under a certain level of anisotropy, such as having a cost function formulated in such a way thatit varies mostly in the directions parallel to the reference axes [24,216]. This is the case depicted in Figure 11b. Sethian and Vladimirsky [215] proposed the use of an algorithm called the Ordered Upwind Method (OUM) to deal with the static HJB equation, the convergence rate of which was demonstrated by Shum et al. [217]. Its main drawback is the increase in computational cost it entails, proportional to the anisotropy existing in the scenario. Shum et al. [31] used HJB for anisotropic path planning, considering energy minimization and stability, and considering the direction and magnitude of slopes. The Fast Sweeping Method (FSM) has also been demonstrated to work with general static HJB equations [218]. It works by visiting all nodes on a grid, following certain directions repeatedly, which means that it demands a high number of iterations. Takei and Tsai [37] used FSM to formulate the HJB equation to comply with turning radius constraints. For the *Eikonal* case, Bak et al. [219] introduced an improvement to FSM to speed up its computation when the cost varies too much, and Detrixhe et al. [220] introduced a parallel version. Jeong and Whitaker [221] proposed an algorithm named the Fast Iterative Method (FIM) to solve the *Eikonal* equation on parallel architectures as well.

### 6.2. Global Optimization

This subcategory contains path planning algorithms that optimize an existing preliminary feasible path. Unlike *Local Optimization* methods, introduced in Section 3.1, *Global Optimization* methods make the resulting path globally optimal in exchange for investing more of a computational load. The approach presented by Ratliff et al. [222], for instance, uses *Sampling-Based* methods such as RRT or PRM as a first step. The second step consists of using gradient optimization techniques to approximate the optimal solution from this feasible path. Van Den Berg et al. [223] also started with a trajectory computed using RRT, to later apply jt to an optimization process based on Differential Dynamic Programming (DP). Plonski et al. [70] used DP to calculate a path in a solar map that dynamically changes, considering that the robot harvests solar energy. Ajanović et al. [224] combined DP with Model Predictive Control (MPC) to calculate energy-minimizing paths. Other techniques include the bang-bang approach [225] and Mixed-Integer Linear Programming (MILP) [226]. Finally, a remarkable approach was proposed by Kogan and Murray [227], who used nonlinear optimization to plan time-optimal paths with a length between 20 and 70 m.

## 7. Summary and Conclusions

Table 3 summarizes the main features of each path planning category according to the classification system proposed in this paper (see Figure 1). It analyzes whether the algorithms require a preliminary model of the environment, whether they are deterministic (i.e., they always provide the same solution given the same initial conditions), whether they can tackle dynamic environments and replan, whether they are optimal and if they are complete (i.e., they always return a path if it is feasible). Given the scope of the final path planning application, some algorithms will be more suitable than others. Moreover, the reach of the planner and the replanning capability, i.e., the capability to deal with updates in the environment information, will determine if an algorithm is more suitable for local planning or global planning. Local planning usually requires fast online computation and this reactivity behavior is required to plan new paths in the presence of environmental data changes. Global planning can even be computed offline and aims to generate paths for long traverses, having a static initial environment available.

*Reactive-Computing*-based algorithms seem suitable for local obstacle avoidance path planning as they are easy and cheap to implement. Furthermore, *Reactive Manoeuvre* methods are a good option for scenarios with high uncertainty or when using a robot with very limited sensing options. *Local Optimization* allows one to even consider kinodynamic constraints with TEB, although they do not ensure completeness. Special attention must be given to both subcategories, in order to avoid falling into a local minimum. *Soft Computing* algorithms produce a path using multiple configurable operators, which can be inspired by nature or can be based on fuzzy rules and/or neural networks. They are suitable for problems involving a large number of variables or problems that are difficult to model, such as in highly dynamic environments. With scenarios containing moving elements, in long-range (global path planning) scenarios, the use of *Evolutionary* methods is adequate. The latest *Artificial Intelligence* methods, including the DL and RL methods, still need to be further studied to obtain solid conclusions, as also remarked by Sun et al. [21]. *Artificial Intelligence* methods based on Fuzzy rules or neural networks can be used for fast *Local Planning* as an alternative to *Reactive Manoeuvre* methods. *C-Space Search* algorithms make use of samples to represent the different configurations of the robot. These samples can be provided beforehand in the form of a graph or they can be dynamically created. *Graph Search* algorithms are suitable for global path planning considering advanced graphs such as visibility graphs or space-lattice graphs, at the expense of investing time into building them (something that is admissible for offline planning). Nevertheless, it scales poorly with problems of high dimensions, which justifies the use of *Sampling-based* algorithms instead. *Sampling-based* algorithms have also proved useful for this kind of manoeuvre and problems with a high number of dimensions. *Optimal Control* algorithms are outstanding for obtaining globally optimal results. *PDE-Solving*-based algorithms depend heavily on the formulated PDE, which can be based on isotropic or anisotropic cost functions and can work with a map model in the form of a grid. *Global Optimization* algorithms have to start with an already-defined path an adapt it according to the robot’s locomotion restrictions. *PDE Solving* algorithms are adequate for the off-line computation of long traverses given low-uncertain static scenarios, as they provide optimal paths without relying on replanning. Finally, it is important to note that all these planners rely on the available information describing the environment and the robot. This information must be modeled as accurately as possible to improve the results of the path planner.

## Figures and Tables

**Table 1:** Main surveys and reviews of global path planning algorithms found in the literature.

	Reactive Computing		Soft Computing		C-Space Search		Optimal Control	
**Publication**	**Reactive** **Manoeuvre**	**Local** **Optimization**	**Evolutionary** **Computation**	**Artificial** **Intelligence**	**Sampling** **Based**	**Graph Search**	**PDE** **Solving**	**Global** **Optimization**
Raja and Pugazhenthi [3] (2012)	Yes	No	Yes	No	No	No	No	No
Nash and Koenig [11] (2013)	No	No	No	No	Yes (RRT and PRM)	Yes (Any-angle and A*)	No	No
Souissi et al. [7] (2013)	Yes (APF)	No	Only mentions	No	Yes	Yes	No	No
Elbanhawi and Simic [12] (2014)	Only mentions	No	Only mentions	Only mentions	Yes	Only mentions	No	No
González et al. [13] (2015)	No	Yes	No	No	Yes	Yes	No	Yes (NLP)
Mac et al. [8] (2016)	Yes	No	Yes	Yes	Yes	No	No	No
Noreen et al. [14] (2016)	No	No	Only mentions	No	Yes	Only mentions	No	No
Injarapu and Gawre [15] (2017)	No	No	Yes (GA)	Yes	No	No	No	No
Ravankar et al. [16] (2018)	No	Yes	No	No	No	No	No	Yes (NLP)
Zhang et al. [4] (2018)	Yes	No	Yes	Yes (ANN)	No	Yes	No	No
Zafar and Mohanta [9] (2018)	Yes (APF)	No	Yes	Yes (ANN)	Yes	Yes	No	No
Costa and Silva [17] (2019)	No	No	Yes (GA)	No	Yes (RRT)	Yes (A*)	No	No
Patle et al. [10] (2019)	Yes (APF)	No	Yes	Yes	Only mentions (PRM)	No	No	No
Gómez et al. [18] (2019)	No	No	No	No	No	No	Yes (eikonal solvers)	No
Campbell et al. [19] (2020)	Yes	No	Yes	Yes	No	No	No	No
Zhang et al. [20] (2020)	Yes	No	Yes	No	Yes	Yes (A* Variants)	Yes (Fast Marching)	Yes (DP)
Sun et al. [21] (2021)	Only mentions (APF, DWA)	No	Only mentions	Yes	Only mentions (RRT and PRM)	Only mentions	No	No
Vagale et al. [6] (2021)	Yes	No	Yes	Yes (DRL)	Yes (RRT and PRM)	No	No	No

**Table 2:** Intelligent models used for some swarm algorithms, together with some of the models.

Individuals	Behaviors
Particles [115,116,117,128]	Best position memory, variable velocity
Fireflies [129]	Brightness atraction
Dragonflies [130]	Hunting and migration
Grasshoppers [131,132]	Attraction and repulsion
Wolves [133,134]	Hierarchy system, hunting for prey
Whales [135,136]	Spiral bubble-nets
Cuckoos [137]	Lévy flights, brood parasitism
Bats [138]	Echolocation
Bacteria [139]	Foraging

**Table 3:** Overview of characteristics of the presented path planning algorithms.

Category	Sub-Category	Preliminary Map Model	Deterministic	Replanning	Optimality	Completeness
Reactive Computing	Reactive Manoeuvre	No [7]	Yes	Yes	Sub-optimal	Not ensured
	Local Optimization	Depends on planner used in first step	Yes	Yes	Sub-optimal	Not ensured
Soft Computing	Evolutionary	Only ACO	No	Yes [3]	Heuristic	Depends (e.g., GA no; PSO, yes) [20]
	Artificial Intelligence	Depends	No	Yes	Heuristic	Not ensured
C-Space Search	Graph Search	Yes [7,11]	Yes [7]	Only incremental	Global restricted to graph	Yes
	Sampling-Based	No	No [7]	Yes	Asymptotical	Probabilistic
Optimal Control	PDE Solving	Yes	Yes	Very rare [212,213,214]	Globally optimal	Yes
	Global Optimization	Depend on planner used in first step	Yes	Yes [3]	Globally optimal	No [20]

## Data Availability

Not applicable.

## Abbreviations

The following abbreviations are used in this manuscript:

ACO	Ant Colony Optimizer
AIT*	Adaptively Informed Tree
APF	Artificial Potential Fields
ABIT*	Advanced Batch Informed Trees
BIT*	Batch Informed Trees
D*	Dynamic A*
DEM	Digital Elevation Map
DL	Deep Learning
DP	Dynamic Programming
DPP	Dynamic Programming Principle
DWA	Dynamic Window Approach
FIM	Fast Iterative Method
FMM	Fast Marching Method
FMT*	Fast Marching Tree
FMS	Fast Marching Square
FSM	Fast Sweeping Method
FM*	Heuristic Fast Marching
GMT*	Group Marching Tree
HJB	Hamilton-Jacobi-Bellman
LPA*	Lifelong Planning A*
MPC	Model Predictive Control
OUM	Ordered Upwind Method
PDE	Partial Derivative Equation
PRM	Probabilistic Roadmap Method
PSO	Particle Swarm Optimizer
RABIT*	Regionally Accelerated Batch Informed Trees
RDT	Rapidly Deterministic Tree
RL	Reinforcement Learning
RRT	Rapidly Random Tree
RRT*	Heuristic Rapidly Random Tree
TEB	Timed Elastic Bands
VFH	Vector Field Histogram
